# The Effect of Fiber Weight Fraction on Tribological Behavior for Glass Fiber Reinforced Polymer

**DOI:** 10.3390/polym17060720

**Published:** 2025-03-09

**Authors:** Corina Birleanu, Razvan Udroiu, Mircea Cioaza, Marius Pustan, Bere Paul, Cristian Vilau

**Affiliations:** 1 MicroNano Systems Laboratory, Mechanical Systems Engineering Department, Technical University from Cluj-Napoca, Blv. Muncii nr. 103-105, 400641 Cluj-Napoca, Romania; corina.barleanu@omt.utcluj.ro (C.B.); marius.pustan@omt.utcluj.ro (M.P.); 2 Manufacturing Engineering Department, Transilvania University of Brasov, Blv. Eroilor Nr. 29, 500036 Brașov, Romania; 3 Manufacturing Engineering Department, Technical University from Cluj-Napoca, 400641 Cluj-Napoca, Romania; 4 Mechanical Engineering Department, Technical University from Cluj-Napoca, 400641 Cluj-Napoca, Romania; cristian.vilau@tcm.utcluj.ro

**Keywords:** glass fiber, tensile strength, friction coefficient, abrasive wear, pin-on-disc tribometer, design of experiments, statistical analysis, GLM method

## Abstract

The tribological performance of Glass Fiber Reinforced Polymer (GFRP) composites is essential for applications in automotive, aerospace, and industrial sectors. This study investigates the effect of fiber weight fraction ratio (wf.) (50%, 65%, and 70%), applied load, and sliding speed on the tribological behavior of twill-woven GFRP using a pin-on-disc tribometer. Experimental trials were carried out to assess the impact of control factors on the coefficient of friction, specific wear rate, and contact temperature. Statistical analyses based on generalized linear models (GLM) method or multi-factor ANOVA, identified the most significant factors and their contributions. Results indicate that sliding speed contributes the highest to COF (46.51%), while fiber wf. primarily influences wear rate (34.15%). The applied load was found to have the strongest impact on contact temperature (39.08%). Furthermore, SEM and EDS analyses reveal dominant wear mechanisms, including abrasive wear and transfer layer formation. This study introduces the novelty of using statistical modeling to optimize GFRP for high-performance tribological applications, providing a more precise and efficient approach to enhancing their properties.

## 1. Introduction

In recent years, the development of next-generation materials has progressed significantly. Traditional materials are increasingly being replaced by composite materials due to their superior properties, including high tensile strength, excellent strength to wf., and low thermal expansion [1]. Moreover, they exhibit full biodegradability through composting processes and do not emit toxic or harmful components [2].

FRP composites have been extensively employed in diverse engineering fields because of their outstanding combination of stiffness and strength relative to weight. Among them, GFRP composites have gained widespread adoption across multiple industries due to their exceptional strength to wf., corrosion resistance, and adaptability to diverse environmental conditions. These materials are extensively used in the automotive, aerospace, and marine sectors, where friction and wear performance significantly influence component longevity and operational efficiency. Additionally, GFRP composites offer highly flexible design solutions, remarkable durability, and cost-effective production and assembly processes [2].

The mechanical characteristics of GFRP composites are influenced by multiple factors, such as the type of fiber and its volume proportion, distribution, orientation, and void content. Furthermore, interfacial bonding characteristics and load transfer mechanisms at the fiber-matrix interface play a critical role in determining the composite’s mechanical behavior [3]. Davallo et al. [4] demonstrated that flexural properties derived from force-deflection data of composites containing 20 wf. and 30 wf. randomly oriented continuous fibers exhibited flexural strength values of 84 MPa and 110 MPa, respectively, with corresponding flexural modules of 7 GPa and 10 GPa.

Experimental studies from the literature suggest that the tensile strength of GFRP composites is greatly influenced by different environmental conditions [3,4]. Abdullah et al. [5] investigated two commercial GFRP composites: chopped glass fiber composites and 0/90 woven fiber-reinforced composites with an unsaturated polyester matrix. Their results revealed that the composite with chopped fibers exhibited higher Young’s modulus, maximum stress, and yield strength compared to the 0/90 composite. Likewise, Wazery et al. [3] observed that tensile strength ranged from 28.25 MPa to 78.83 MPa, flexural strength varied between 44.65 MPa and 119.23 MPa, and impact energy at room temperature fluctuated between 3.5 Joules and 6.5 Joules, depending on the glass fiber content (15 wt.% to 60 wt.%). These studies underscore the significant enhancement in mechanical properties, such as tensile and flexural strength, conferred by glass fiber reinforcement in polyester resin matrices [3].

Beyond mechanical performance, FRP composites also represent a vital class of tribo-materials. In applications involving sliding contact, understanding and optimizing the tribomechanical behavior of GFRP composites is essential for improving performance and durability. Tribological performance is influenced by multiple parameters, including operating conditions, fiber and matrix characteristics, manufacturing processes, interfacial properties, and contact conditions [6]. Numerous studies have been carried out in this area to improve the durability and frictional performance of these materials.

Zhao et al. [7,8] and Karsli et al. [9] have investigated various sliding conditions, such as dry adhesion sliding, abrasion, and erosion. Their findings suggest that wear performance under different sliding conditions is not necessarily correlated due to distinct wear mechanisms governing each case. The wear and friction characteristics of GFRP composites are mainly influenced by fiber weight fraction, applied force, and sliding velocity.

These factors influence critical indicators like the coefficient of friction (COF), specific wear rate, and contact temperature. Experimental studies indicate that optimizing these parameters can significantly improve wear resistance and friction efficiency, broadening the application scope of these advanced materials [10,11,12,13,14,15].

However, optimizing tribomechanical parameters remains challenging due to the complex interplay between material properties and operational conditions. Traditional experimental approaches often fail to fully capture these intricate relationships, necessitating the application of advanced statistical models for comprehensive analysis. Techniques such as analysis of variance (ANOVA) and Grey Relational Analysis (GRA) have been successfully employed to optimize the tribomechanical behavior of composite materials [15,16,17,18,19,20]. Also, in [10], based on the ELECTRE decision-making method, the tribological parameters have been optimized by assigning weighted factors to the coefficient of friction and specific wear rate. However, the effect of control factors on tribological parameters was not analyzed. The type of reinforcement and the tribological system strongly influence the wear rate, friction coefficient, and wear mechanisms, making them crucial factors in composite selection for specific applications [6]. From the literature survey, few research studies deeply investigated the effect of control factors on tribological properties of different composite materials.

Zaghloul et al. [21] found that glass fiber reinforcement in thermoplastic polymers, particularly at a 33% volume fraction, significantly improved wear resistance. However, increased fiber volume fractions inversely affected wear resistance, highlighting the complex relationship between mechanical properties and tribological performance. GFRP composites exhibit improved tensile strength with increased fiber volume fractions, with optimal performance at 25% to 50% volume fractions [21].

Lower friction coefficients are observed at optimal fiber orientations and volume fractions, enhancing the material’s suitability for applications like bearings and gears [22].

Abdurohman et al. [23] suggested that different manufacturing processes (e.g., vacuum infusion, hand lay-up) affect the compressive and shear properties of GFRP, with vacuum infusion yielding superior mechanical performance.

Ali et al. [24,25] focus on the tribological properties of glass fiber reinforced polymer composites (GFRP), highlighting that different thicknesses of GFRP exhibit varying wear rates and sliding characteristics, with thicker samples demonstrating superior performance in mechanical tests and real-world applications.

The number of research papers published in the last two decades focusing on the wear of materials at different fiber volumetric fractions has been limited. The scarcity of studies in previous years has been a key motivation for evaluating the wear performance of reinforced polymers by adjusting fiber volume fractions. Considering the research gaps highlighted in the review article by Zaghloul et al. [21], this study aims to optimize the tribological parameters of a pin-on-disc friction system, where the pin is made of steel, and the disc consists of a GFRP composite. The experimental investigation will analyze the effects of fiber wf. (50%, 65%, and 70% wf.), applied load (10 N, 20 N, and 30 N), and sliding speed (0.1 m/s, 0.25 m/s, and 0.36 m/s) over 120 min. Statistical techniques, based on generalized linear models (GLM) method or multi-factor ANOVA, have been used to identify the most influential factors and their contributions to tribological performance. By optimizing these factors, this study seeks to offer meaningful contributions to the design and advancement of high-performance composite materials for tribological applications. SEM and EDS analyses identify key wear mechanisms, such as abrasive wear and the formation of a transfer layer.

## 2. Materials and Methods

### 2.1. Materials and Samples Manufacturing

Three testing samples were manufactured from glass fiber reinforced polymer composite materials at different fiber wf. of 50%, 65%, and 70%. A thermosetting polymer matrix was chosen, specifically the MGS LR135 epoxy resin, which was combined with the MGS LH136 hardener at a mixing ratio of 100:35 ±2 g. Both components were supplied by Hexion (Duisburg, Germany). The epoxy resin MGS LR135, used in manufacturing our composite, is supplied by the manufacturer Hexion, and its material properties, including thermal behavior, are well-documented by the supplier. The heat treatment in the GFRP curing process, according to the tested cycle, includes a curing stage of 3 h at 120 °C. As is known, the resin withstands temperatures up to 10 degrees lower than the temperature it was exposed to during the heat treatment in the curing process, without its properties being affected. According to experimental results, the temperature during the tribological testing process did not reach this value. In this study, we relied on the resin manufacturer’s documentation, which specifies the resin’s heat treatment and curing procedure for certain applications. The reinforcement of the composite material was achieved using 270 g/m^2^ twill-woven glass fiber fabrics to ensure mechanical stability and performance.

The production of GFRP plates with fiber wf. of 50%, 65%, and 70% adhered to a strictly regulated process to ensure uniformity and high-quality test specimens. The primary impregnation method utilized was the hand lay-up technique, wherein fiber layers were manually arranged and impregnated with epoxy resin. A total of six layers of twill-woven glass fiber fabric were used, each measuring 500 mm × 300 mm. The fibers were thoroughly impregnated with the resin matrix to protect them and facilitate effective load transfer. After applying the resin and fabric layers simultaneously, the entire surface was manually rolled to remove any air bubbles trapped between the overlapping layers.

**Figure 1:**
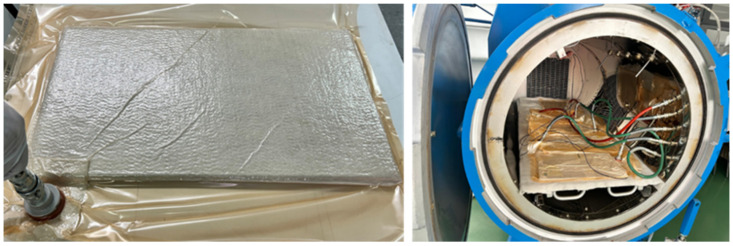
Vacuum bagging technique of the laminated sample and autoclave curing process.

In the subsequent stage, the GFRP material was covered with a release film, and a breather being placed inside a vacuum bag, as shown in Figure 1. For the curing procedure, an autoclave manufactured by Maroso (Maroso SRL, Veneto, Italy) was employed. This equipment enabled precise control of temperature and pressure during the curing process. The vacuum bag was subjected to a vacuum pressure of −0.9 bar during all curing cycle steps. The autoclave parameters were, Step 1, temperature increased at 80 °C, pressure 4 bars 30 min; Step 2, temperature increases at 120 °C pressure at 4 bars, 30 min; Step 3, temperature 120° C, 180 min, pressure 4 Bars; and Step 4, cooling procedure at 25 °C, 120 min.

The vacuum bagging process used in this study was carefully controlled to minimize defects and ensure uniform resin impregnation. However, as with any composite manufacturing process, a certain degree of porosity may be present. The wet resin impregnation method was employed, followed by vacuum bagging and autoclave curing. From a practical standpoint, it is impossible to achieve a completely pore-free structure using this method, which is considered one of the most effective in minimizing porosity. Any porosity that does occur is not present within the bulk material structure but manifests as a very slight surface porosity on the composite plates. When using pre-impregnated material, the structures obtained with these technologies may exhibit lower porosity. This is due to the reinforcement weight fraction ratio imposed by manufacturing companies. In this study, the main challenge was to achieve a controlled reinforcement weight fraction ratio at the initially established values while ensuring that the resin was not expelled from the reinforcement material under high pressures (50 tons/m) during the polymerization process.

As a result of this process, multiple composite plates were obtained, each measuring 500 mm × 300 mm × 2 mm, with reinforcement levels of 70%, 65%, and 50% (±0.5%). During the post-processing stage, after curing, the composite parts may require cutting, finishing, or mechanical processing to achieve the final dimensions and specifications. Any defects identified can be rectified through additional application of resin and reinforcing materials.

**Figure 2:**
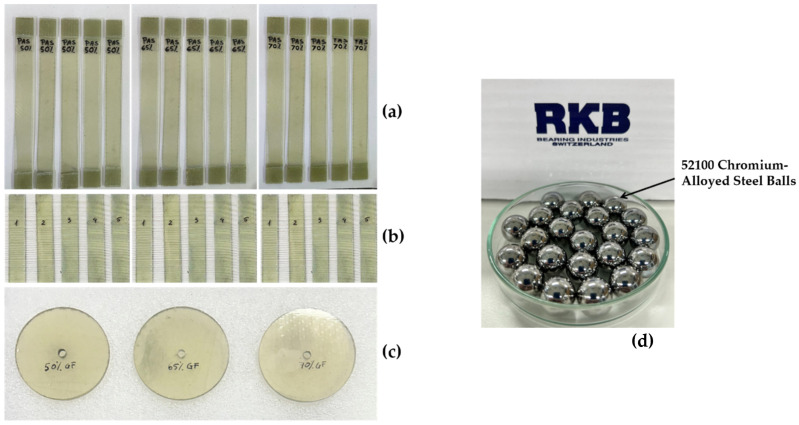
Specimens of GFRP composites (50%, 65%, and 70% wf.) used for: (**a**) mechanical tensile tests; (**b**) mechanical flexure tests; (**c**) tribological tests; and (**d**) 52100-chromium-alloyed steel balls used in tribological tests.

The prepared test specimens included:

- Tribological test discs: 50 mm in diameter and 2 mm in thickness, used for wear resistance analysis (Figure 2a);
- Tensile test specimens: 250 × 25 × 2 mm, prepared according to ASTM D3039-17 standard [26] (Figure 2b);
- Bending test specimens: 80 × 13 × 2 mm, prepared in accordance with the ASTM D7264D standard (Figure 2c).

Compliance with these standards ensured consistent sample quality, enabling accurate and reproducible mechanical testing. In the initial phase, the polymeric semi-couples manufactured for experimental testing are presented in Table 1 and Figure 2a,b.

Bearing balls made of 52100-chromium-alloyed steel (RKB Bearing Industries Group, Balerna, Switzerland) measuring 12.7 mm in diameter were utilized during the wear tests based on the recommendations of the ASTM A295 standard, (Figure 2d). Bearing balls were used in the wear tests as counter pieces against the circular composite material specimens (discs).

### 2.2. Mechanical Tests

The analysis of experimental data related to the tribological and mechanical testing of FRP materials involves several systematic steps, from processing primary data to interpreting and comparing the results. The mechanical testing of GFRP specimens was conducted in accordance with ISO 527-1:2019 (standard for tensile testing) and ISO 178:2003 (standard for flexural testing). The ISO standards correspond to ASTM D638-14 (Standard Test Method for Tensile Properties of Plastics) and ASTM D790-03 (Standard Test Methods for Flexural Properties of both Unreinforced and Reinforced Plastics).

The mechanical testing was conducted in the Accredited Mechanical Testing Laboratory of the Department of Mechanical Engineering, Technical University of Cluj-Napoca, using a mechanical testing machine, specifically the Instron 8801 (Instron, Norwood, MA, USA), with a maximum load capacity of 100 kN. The equipment automatically records the loading process in the form of a stress-strain curve, which represents the applied stress in the specimen and the corresponding strain. Each test involved five specimens. The applied load was controlled at a crosshead speed of 2 mm/min until specimen failure, under ambient conditions of 18 °C temperature and 50% relative humidity. For flexural testing, the specimens (Figure 2b) were evaluated using a three-point bending test with a universal testing machine, Instron 3366-10 kN (Instron, Norwood, MA, USA). The parameters set for the flexural tests were identical to those used for the tensile tests.

### 2.3. Wear Tests

Tribological testing is crucial for evaluating the behavior of materials under conditions similar to their actual operational environment, enabling the understanding and optimization of performance in industrial applications. In this context, the tests performed on GFRP specimens in contact with steel balls aimed at characterizing the composite material’s behavior under specific dry friction conditions. The experimental procedures for these tests follow established standards, including ASTM G99 (Wear Testing with a Pin-on-Disk Apparatus) and ASTM F732 (Wear Testing of Polymeric Materials).

The study of surface interactions and behaviors in relative motion is highly dependent on operating conditions, which play a fundamental role in evaluating the performance of systems and materials. The establishment and control of operating conditions, as detailed in Table 2, are essential for ensuring accurate and relevant results. Tribological variables such as the coefficient of friction and wear rate can be significantly influenced by working parameters and environmental conditions during the experiment.

Prior to testing, specimen preparation involved careful cleaning with technical alcohol and weighing using an analytical balance with 0.1 mg precision. After each test, both the steel balls and composite discs were replaced with new ones to ensure consistency and avoid cross-contamination. By establishing and maintaining appropriate and consistent operating conditions, reliable and reproducible data can be obtained, enabling the evaluation and comparison of different materials or systems across various operating scenarios. Moreover, this approach allows for the identification of key factors influencing tribological behavior and the development of effective strategies for improving the performance and durability of studied materials. During the experimental trials, operating conditions were strictly monitored and controlled to guarantee precision and reliability of the obtained data, allowing for meaningful and applicable conclusions to be drawn from the results.

The tribological analysis focused on abrasive wear evaluation, material transfer mechanisms, and thermal stability, with continuous monitoring of humidity, chamber temperature, and local frictional temperature. Additionally, the coefficient of friction was continuously recorded, and wear assessments were performed. Surface wear images were captured, test parameters were documented, and material transfer was analyzed.

Friction and wear tests were conducted using a pin-on-disc tribometer under the conditions outlined in Table 2. To improve the reliability of the results, each experiment was repeated five times, with the reported values representing the average measurements. The dispersion of results was highlighted using error bars, representing the corresponding standard deviation.

For each test, a new ball and disc were utilized, and prior to experimentation, they were cleaned with ethanol and then dried in a controlled environment. Throughout the test, temperature, worn surface, and friction force were continuously monitored. Coefficients of variation (CV) of the tribo-mechanical parameters were calculated based on standard deviation and mean values. The coefficient of variation is a measure of spread that describes the variation in the data relative to the mean. CV coefficients less than 10% were determined, which confirm the repeatability of the experiments.

The test was conducted for 120 min, during which the coefficient of friction was recorded in real time, capturing both the initial run-in phase and the steady-state friction regime. The temperature generated during the experiments was also continuously monitored. After a specified number of cycles, the test was temporarily halted to perform a topographic surface analysis, allowing for wear quantification and assessment of surface roughness evolution.

To achieve a more comprehensive understanding of the wear phenomenon and material removal mechanisms, 3D optical microscopy (OM) was employed to examine the worn surfaces of the ball and the running surface of the disc after each test. Surface modifications were assessed using the 3D Nano Focus optical microscope (NanoFocus AG, Oberhausen, Germany), which incorporates advanced μsurf technology for high-precision 3D surface measurements [16].

Accurate measurement of the wear track is functionally essential. The profilometric measurement method is particularly useful but is susceptible to measurement errors. Factors such as human judgment and measurement techniques significantly influence the accuracy of wear track evaluation. The best practice involves performing a complete surface measurement of the wear track. The number of scans represents the most critical factor in measuring the wear track in the pin-on-disc configuration, with a higher number of scans being required for non-homogeneous wear tracks. In this study, eight scans were performed on each disc at a 45° angle between them, allowing for a more precise estimation of volumetric wear, which led to a reduction in uncertainty. To validate the measurements, in addition to μsurf technology, the discs were also scanned using the wear modulus of the Tribometer TRB^3^ (Anton Paar GmbH, Graz, Austria). The modelization and tribometer software share the same InstrumX user interface with modular technology, which enables a complete integration of various data in a single document including tribometer measurements and modelization, image management, statistics, and reporting functions.

The wear rate (*K*, mm^3^/Nm) was determined using Equation (1) [16]:

(1) $$K_{disc}=\frac{V_{disk}}{L_{sliding}\cdot F};\;K_{ball}=\frac{V_{ball}}{L_{sliding}\cdot F}$$

where it is noted that:

- *V_disk_*—wear volume of the disc (mm^3^);
- *V_ball_*—wear volume of the ball (mm^3^);
- *F*—normal force (N);
- *L_sliding_*—total sliding distance (m);
- *K_disk_*, *K_ball_*—wear factors of the disc and ball (mm^3^/Nm).

The wear volume of the specimens was calculated by measuring the width and depth of the wear tracks using advanced μsurf technology. These calculations were derived from empirical mathematical equations, assuming the ideal geometric accuracy of the ball as a model for the wear marks.

Additionally, an infrared thermal camera, FLIR E5xt (Teledyne FLIR Company, Wilsonville, OR, USA), equipped with MSX and Wi-Fi technology, within a temperature range of −20 °C to +400 °C, a 160 × 120-pixel resolution, and a 9 Hz refresh rate, was used to monitor temperature variations during the tests.

### 2.4. Design of the Experiments and Statistical Method

The tribological experimental plan was designed to assess the impact of three control factors—fiber weight fraction (wf.), applied force (F), and sliding speed (v)—on three response variables: coefficient of friction (COF), specific wear rate (K), and temperature (T). Each control factor was evaluated at three distinct levels, as detailed in Table 3.

A general full factorial design with 27 factor combinations was implemented to examine the effects of control factors on target variables. Statistical analysis was conducted using Generalized Linear Models (GLM) or multi-factor ANOVA in Minitab 19 software (Coventry, UK). The percentage contribution ratio (PC%) of individual factors and their interactions was determined. Significant control factors were identified from the ANOVA tables based on F-values and p-statistical parameters. Graphical methods, including main effects plots, interaction effects plots, and interval plots of target variables against control factors, were utilized for data visualization. Lastly, ANOVA assumptions were verified to ensure the validity of the analysis.

### 2.5. Morphological Analysis

The JEOL JSM-5600LV Scanning Electron Microscope (SEM), manufactured by JEOL Ltd., Tokyo, Japan, analyzes samples in both high and low vacuum modes, enabling the investigation of conductive and non-conductive materials. Equipped with a tungsten filament electron gun, secondary electron (SE), and backscattered electron (BSE) detectors, it provides high-resolution imaging and compositional contrast.

For elemental analysis, the ULTIM MAX 65 Energy Dispersive X-ray Spectroscopy (EDX) detector from Oxford Instruments, High Wycombe, UK, is used. Featuring Aztec 4.2 software, it ensures precise spectral acquisition, real-time element identification, and high-sensitivity mapping. The SEM-EDX system supports microstructural and compositional studies across various scientific and industrial applications.

## 3. Results and Discussion

### 3.1. Results of Mechanical Testing

For mechanical testing, five specimens were prepared for each type of GFRP and subsequently subjected to tensile and flexural testing using the standardized testing methods described above. The mean values of the mechanical properties, along with their standard deviations for all tested specimens, are summarized in Table 4 and Table 5. Specifically, the results of the tensile tests are detailed in Table 4. Flexural tests are used to evaluate the material’s resistance to forces acting perpendicular to its length, measuring failure mode and deformation under bending stresses, and the results are shown in Table 5.

**Figure 3:**
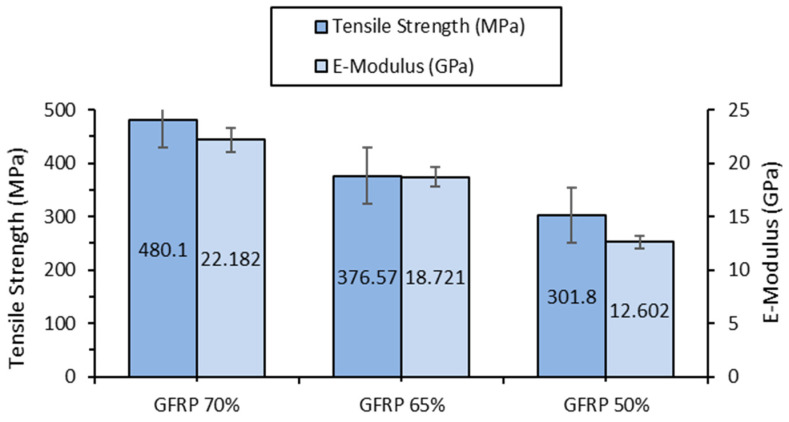
Tensile strength and elastic modulus of GFRP 70%, GFRP 65%, and GFRP 50% specimens.

The increase in glass fiber content significantly improves tensile strength: GFRP 70% exhibits 59% higher tensile strength compared to GFRP 50%. The elastic modulus also increases, indicating higher stiffness. Figure 3 illustrates the variation in tensile strength and elastic modulus for GFRP specimens with different fiber content (70%, 65%, and 50%), highlighting the direct correlation between increased fiber reinforcement and enhanced mechanical performance, particularly in terms of strength and stiffness.

**Figure 4:**
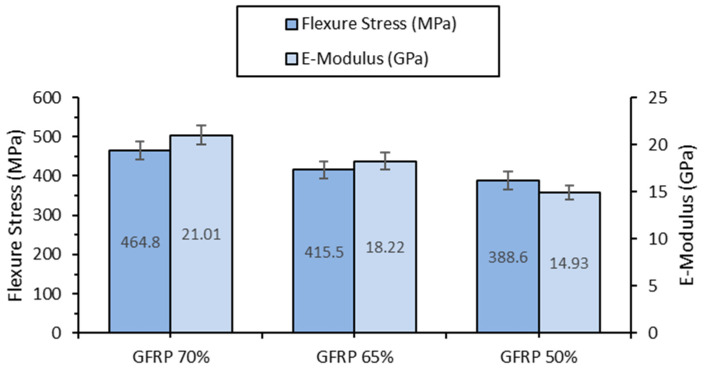
Flexural strength and elastic modulus of GFRP 70%, GFRP 65%, and GFRP 50% specimens.

Flexural tests confirm similar trends observed in tensile testing, demonstrating an increase in resistance to forces acting perpendicular to the specimen’s length. GFRP 70% reaches a flexural strength of 464.8 MPa, approximately 19.6% higher than that of GFRP 50%, which exhibits a strength of 388.6 MPa. The flexural elastic modulus follows the same trend (Figure 4 and Table 4), reaching 21,009 MPa for GFRP 70%, a 40.7% increase compared to GFRP 50%. A notable observation is the variation in flexural strain, where GFRP 50% shows the highest deformation (3.8%), while GFRP 65% exhibits the lowest value (2.4%), indicating a possible nonlinear influence of fiber content on flexural behavior.

Overall, increasing the fiber weight fraction significantly enhances the strength and stiffness of the material, enhancing its suitability for applications that demand a high load-bearing capacity. However, as fiber becomes more dominant in the polymer matrix, ductility may decrease, potentially affecting tribological behavior, particularly in terms of wear particle formation and friction coefficient. These findings suggest that optimizing the tribological and mechanical performance of GFRP composites requires balancing strength, stiffness, and deformation capacity to achieve the best overall performance.

### 3.2. Tribological Testing Results

Tribological testing results provide essential data for determining the coefficient of friction, wear rate, and understanding the degradation mechanisms of the fiber-reinforced composite material in relation to the contact material of the ball.

Throughout the 135 experimentally validated tests conducted for the friction pair, special attention was given to strict control and monitoring of the operating conditions. This rigorous approach ensured the precision and significance of the acquired data, allowing meaningful and applicable conclusions to be drawn from the experimental results. The results of the tribological analysis are summarized in Table 6, highlighting the control factors (fiber weight fraction, applied force, and sliding speed) and the target parameters (coefficient of friction, specific wear rate, and temperature).

The mass reduction of the chromium-alloyed steel balls was negligible, whereas for the disc, the measured weight loss across all experimental tests ranged from 0.001 to 0.030 g, increasing with both sliding speed and applied force. Consequently, the wear rate (*K*, mm^3^/Nm) was determined using the results obtained through 2D Optical Scanning.

**Figure 5:**
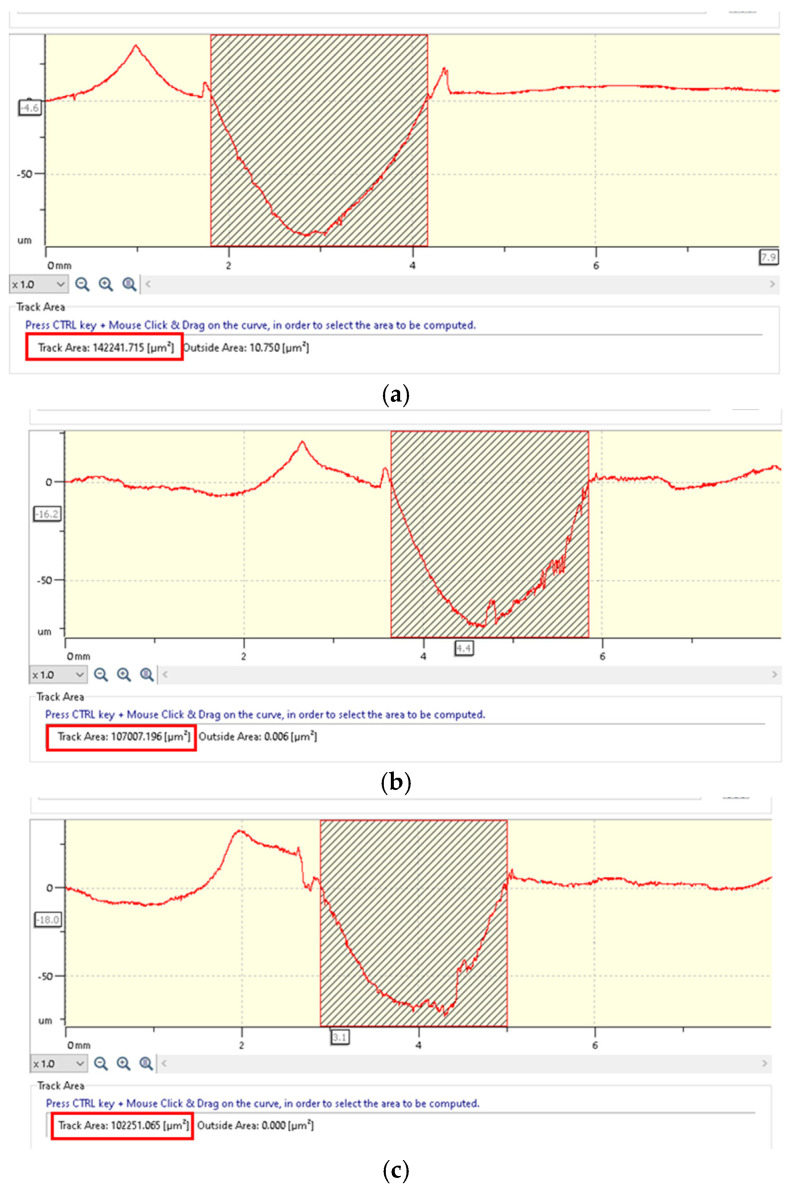
(**a**) A 2D profilometry measurement of wear tracks of the GFRP50 wf. specimen after the dry friction test with F = 30 N, v = 0.25 m/s, duration of 120 min, in contact with a chromium-alloyed steel ball (**left**) and 3D optical profilometry images (**right**); (**b**) similar images for GFRP65 wf.; (**c**) similar images for GFRP70 wf.

Figure 5a–c shows the 2D optical images of the wear track profiles obtained on the GFRP discs in contact with the 52100-steel ball. Based on the experimental profilometry findings, Table 6 presents the wear rate range of the composite disc for three different sliding speeds and corresponding normal values of the applied force.

The minimal wear (Figure 5) observed on the 52100-chromium-alloyed steel balls when in contact with GFRP discs of varying fiber weight fractions (50%, 65%, and 70%) can be attributed to several key factors related to the material properties and tribological interactions. The 52100-steel is a high-carbon, chromium-alloyed bearing steel with excellent wear resistance and high hardness (60–64 HRC), which significantly limits material removal due to abrasive contact. Given that GFRP is significantly softer than steel, the interaction results in minimal wear on the steel balls, as the primary material loss occurs within the polymeric matrix of the composite rather than on the metallic counter face.

### 3.3. Results of Statistical Analysis

The key findings of the statistical analysis for the coefficient of friction, specific wear rate, and temperature are presented in Table 7. A control factor was considered to have a significant influence on the target parameters when the p-value was lower than the significance threshold of 0.05.

The most significant percentage contribution ratios on the coefficient of friction were 46.51% obtained for sliding speed, 32.51% for applied force to sliding speed interaction, and 11% for glass fiber wf. ratio, as is shown in Table 7. The applied load factor and the other two interactions between factors had lower percentage contributions to the coefficient of friction.

This behavior can be explained by the complex interplay of tribological mechanisms that govern friction behavior in glass fiber-reinforced polymer (GFRP) composites under different operating conditions.

The effect of applied load on COF is primarily related to the contact pressure at the interface between the 52100-chromium-alloyed steel ball and the GFRP composite. At lower loads, the contact pressure remains moderate, resulting in stable friction behavior. However, as the load increases, localized deformation at the interface intensifies, leading to fiber-matrix detachment, increased material transfer, and potential modifications in the surface topography. These effects can either increase or decrease COF, depending on the extent of the deformation and wear mechanisms involved.

Sliding speed also plays a critical role in COF variation. At lower speeds, friction remains relatively high due to limited thermal effects. As the sliding speed increases, frictional heating causes localized softening of the polymer matrix, reducing adhesion and lowering the COF. However, at very high speeds, excessive heat accumulation may degrade the polymer surface, potentially increasing COF due to surface roughening or instability in the wear mechanism.

The interaction between load and sliding speed emerges from their combined influence on surface deformation, material transfer, and thermal effects. At high loads and low speeds, stronger adhesive interactions increase COF due to intensified fiber-matrix detachment. As the speed increases under high load conditions, frictional heating facilitates the formation of a polymeric transfer layer, reducing direct contact and lowering COF. However, at high loads and very high speeds, excessive heat buildup can degrade the composite surface, leading to unstable friction behavior. In contrast, low loads combined with high speeds result in lower contact pressure, reducing heat generation and stabilizing COF.

These findings align with our SEM and EDS analyses, which indicate that increased load and sliding speed alter wear mechanisms by affecting fiber exposure, polymer matrix softening, and transfer layer formation. The observed trends further support the explanation of this significant interaction effect.

The glass fiber weight fraction (wf.) exhibited the most significant impact on the specific wear rate, contributing 34.15%, followed by the applied load at 21.87% and the sliding speed at 14.11%, as detailed in Table 7. The interactions among these factors were not statistically significant, as their p-values exceeded the 0.05 significance threshold, as shown in Table 7.

The applied load and the interaction between glass fiber weight fraction (wf.) and sliding speed were the most influential factors affecting temperature, contributing 39.08% and 27.24%, respectively. In contrast, the individual effects of wf. and sliding speed, along with other factor interactions, had lower contributions to temperature, as shown in Table 7.

The graphical results of statistical analysis for all three targets (COF, K, and T) are explained based on the main effects plot, interaction plot and interval plot.

**Figure 6:**
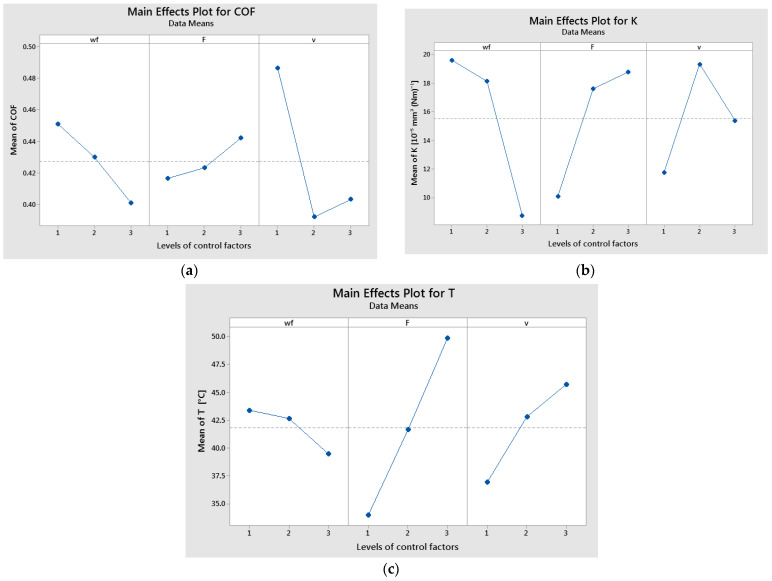
Main effects plot for: (**a**) coefficient of friction; (**b**) specific wear rate; and (**c**) temperature.

The maximum average values of COF were obtained for wf. 50%, force at level 3 (30 N) and sliding velocity at level 1 (0.1 m/s), as shown in Figure 6a. It was observed that there was a decreasing trend for COF with increasing wf., an increasing trend for COF with increasing F, and a large decrease in COF from level 1 to level 2 of v, followed by a small increase. The lowest mean values of COF were obtained for wf. = 70%, F = 10 N, and v = 0.25 m/s.

The main effects plot results indicate that the highest mean values of K were observed for a glass fiber weight fraction (wf.) at level 1 (50%), an applied force at level 3 (30 N), and a sliding speed at level 2 (0.25 m/s), as illustrated in Figure 6b. The specific wear rate decreases with an increase in glass weight fraction. The specific wear rate raises with increasing load applied. It was observed that there was a large increase in K from level 1 (0.1 m/s) to level 2 (0.25 m/s) of v, followed by a small decrease to level 3 (0.36 m/s).

The primary effects on temperature were identified for the glass fiber weight fraction (wf.) at level 1 (50%), the applied force at level 3 (30 N), and the sliding speed at level 3 (0.36 m/s), as illustrated in Figure 6c. Additionally, it was observed that temperature decreased as the glass fiber weight fraction increased.

**Figure 7:**
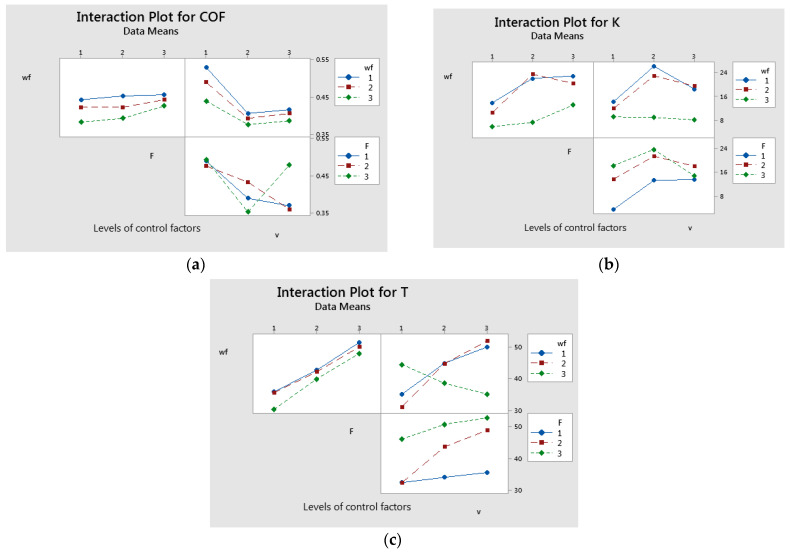
Interaction effects plot for: (**a**) coefficient of friction; (**b**) specific wear rate; and (**c**) temperature.

The interaction between applied force (F) and sliding speed (v) had a significant effect on the coefficient of friction, as illustrated in Figure 7a and confirmed in Table 7. Minimal interactions between factors were observed for the specific wear rate, as shown in Figure 7b. Additionally, the interaction between glass fiber weight fraction (wf.) and sliding speed (v) had a notable impact on temperature, as depicted in Figure 7c.

**Figure 8:**
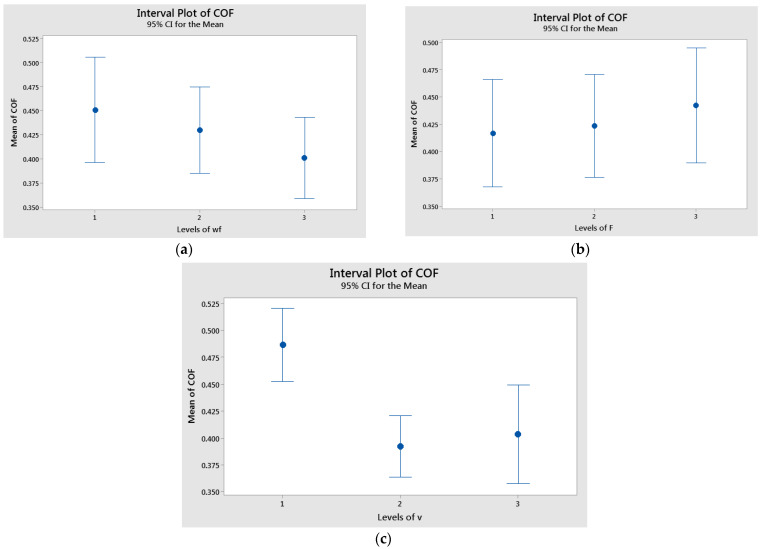
Interval plot of the coefficient of friction (COF) factor with: (**a**) wf.; (**b**) F; and (**c**) v. Individual standard deviations were used to calculate the interval plot. Bars are standard errors of the mean.

The interval plots with standard error bars for each factor in relation to the coefficient of friction are presented in Figure 8. The difference between the means for coefficient of friction in v, were significant because the interval bar for level 1 (v = 0.1 m/s) and the interval bars for level 2 (v = 0.25 m/s) and 3 (v = 0.36 m/s) did not overlap (Figure 8c). The differences for glass weight fraction and applied force were probably not significant because all the interval bars have over-lapped (Figure 8a,b).

**Figure 9:**
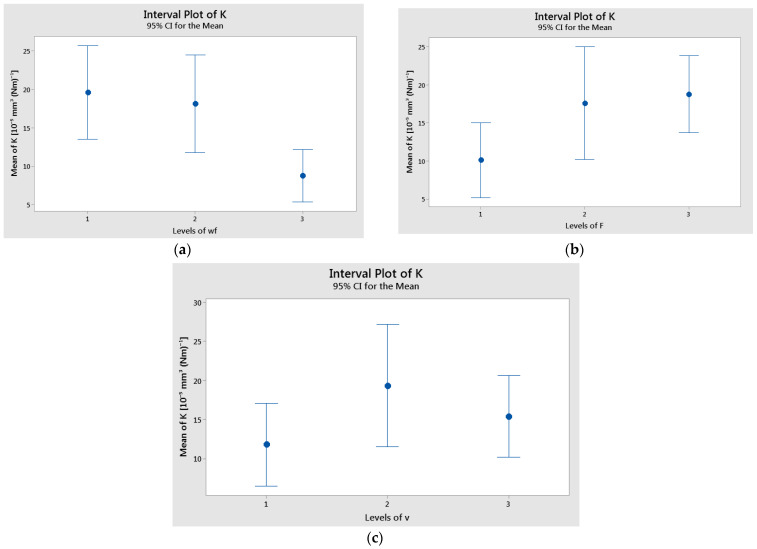
Interval plot of the specific wear rate (K) factor with: (**a**) wf.; (**b**) F; and (**c**) v. Individual standard deviations were used to calculate the interval plot. Bars are standard errors of the mean.

**Figure 10:**
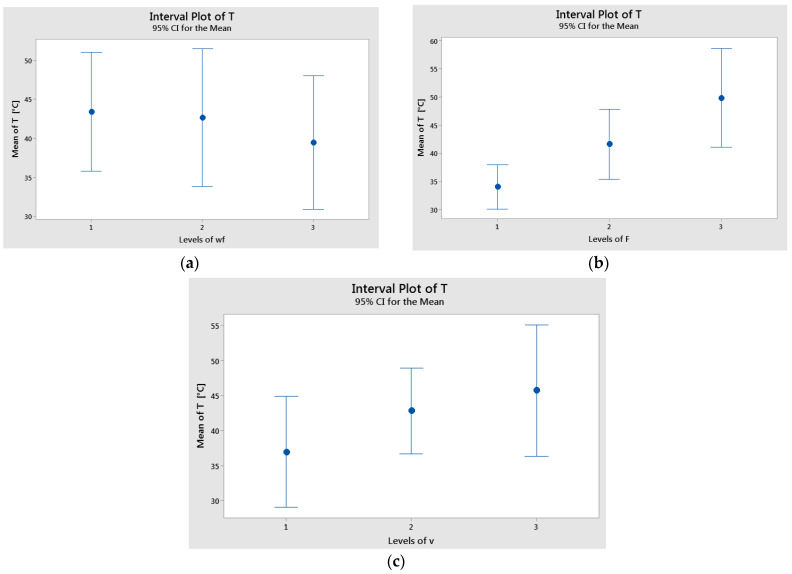
Interval plot of the temperature (T) factor with: (**a**) wf.; (**b**) F; and (**c**) v. Individual standard deviations were used to calculate the interval plot. Bars are standard errors of the mean.

The interval plots of factors versus specific wear rate are shown in Figure 9. The lowest mean values of K were recorded for wf. = 70% (level 3), F = 10 N (level 1), and v = 0.1 m/s (level 2). Similarly, the interval plots of each factor versus temperature are illustrated in Figure 10. The lowest mean temperature values were observed for wf. = 70%, F = 10 N, and v = 0.1 m/s. The difference in temperature means for sliding speed (v) was statistically significant, as indicated by the non-overlapping interval bars between levels 1 and 3.

**Figure 11:**
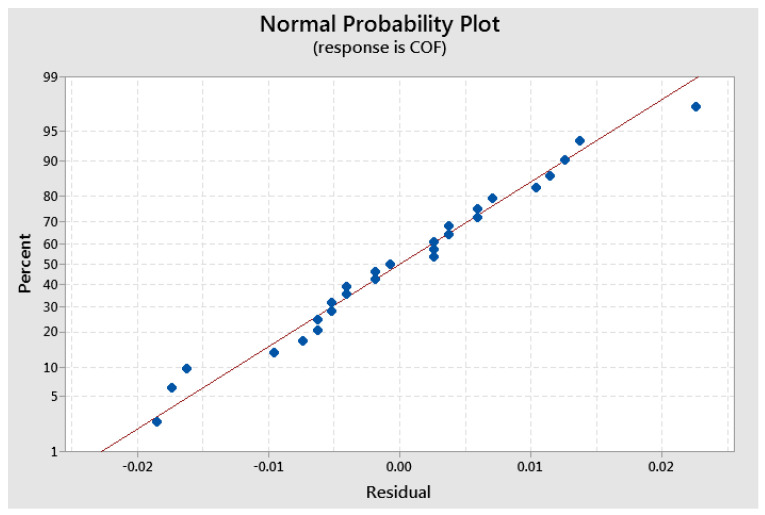
Normal probability plots of residuals for coefficient of friction (COF).

Normal probability plots were obtained for residuals of COF, K, and T, that confirmed the generalized linear models were adequacy [27,28,29,30]. Figure 11 shows the normal probability plot for COF. Similar distributions were obtained for K and T.

### 3.4. Results of the Morphological Analysis

Based on the results obtained from the statistical analysis, a thorough investigation of the worn surface and an SEM analysis were necessary for a complete characterization of the wear mechanism. Thus, after 120 min, the following investigations were performed: optical microscopic investigations of the appearance of the GFRP disc and the steel ball used in the test, as well as SEM and EDS investigations of the worn surfaces of the GFRP samples (50%, 65%, and 70% wf.).

**Figure 12:**
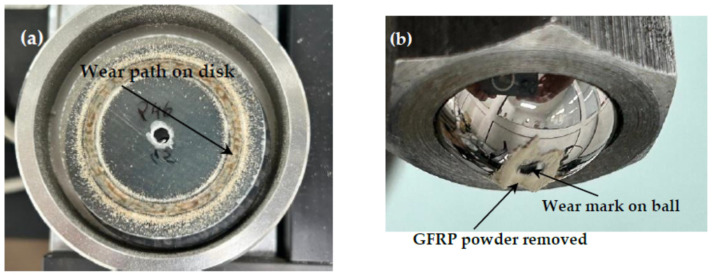
Aspects of wear marks on (**a**) the GFRP disc and (**b**) the steel ball, 52100.

After a 120 min test interval, the GFRP disc and the steel ball subjected to the test parameters look as in Figure 12. Around the contact area of the ball with the disc, material removed by abrasion from the glass fiber reinforced polymer composite disc can be observed.

Various factors, including sliding speed, significantly influence the wear rate (K) in the GFRP/chromium-alloyed steel tribo-system. In general, an overall decrease in the wear rate is observed as sliding speed increases. A possible explanation for this trend is the effect of sliding speed on the development of a transfer layer. At low sliding speeds, the interaction between the ball and the disc results in the accumulation of wear particles, promoting abrasive wear and increasing the wear rate. However, as sliding speed increases, wear particles are partially displaced from the contact area and partially redeposited onto the counterface surface, aiding in the formation of a transfer layer that helps reduce the wear rate.

The wear rate shows a direct increase with sliding duration for all tested specimens. Additionally, higher glass fiber content correlates with a lower wear rate, while composites with lower fiber content experience an increased wear rate. These findings are consistent with the conclusions of Kukureka et al. [19,20], who studied rolling-sliding contact in a similar material system.

The GFRP 50 wt.% specimen demonstrated higher sensitivity to sliding speed compared to composites with greater fiber content, especially under a normal load of 10 N. This wear pattern is attributed to surface property modifications as the glass fiber content decreases in the polymer matrix. A notable difference emerges when the normal load increases to 30 N and the sliding speed reaches approximately 0.25 m/s, especially for the GFRP 50 wf. specimen. At this higher load, the rougher metallic surface asperities of the steel ball deform the polymeric surface, leading to brinelling effects, micro-cutting, and the development of abrasive wear tracks. The observed wear is attributed to the abrasive action of metallic asperities, which is further intensified by the interaction with fragmented glass fiber debris.

**Figure 13:**
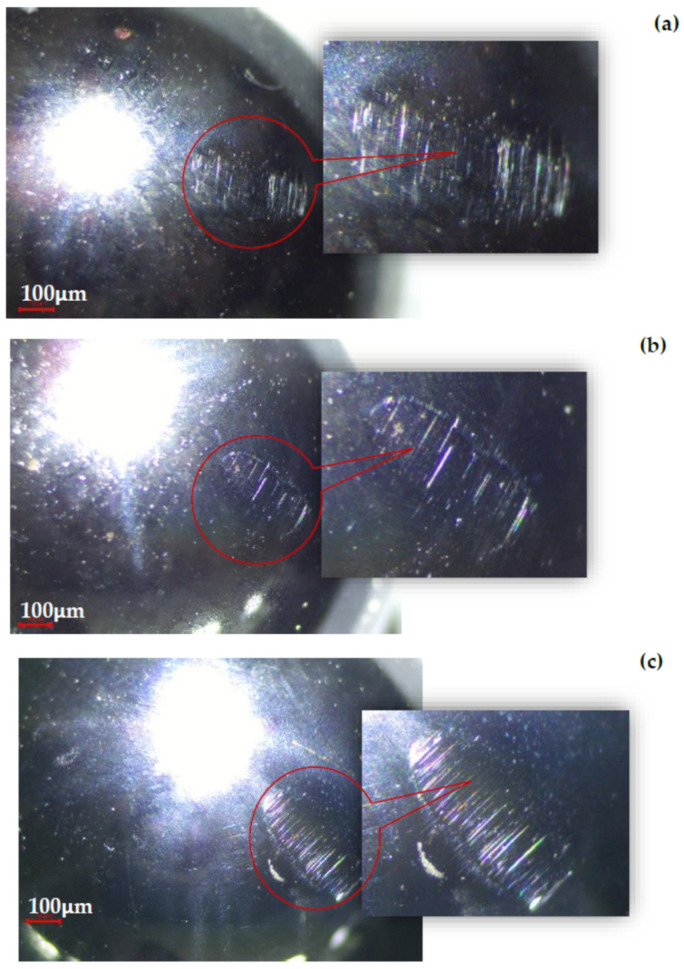
Wear marks on the chromium-alloyed steel ball at a sliding speed of 0.25 m/s under a 30 N load, after sliding on: (**a**) GFRP50 wf. specimen; (**b**) GFRP65 wf. specimen and (**c**) GFRP70 wf.

Glass fibers demonstrate abrasive and lubricating effects on the sliding surface, significantly contributing to friction reduction, especially during the running-in period. This dual behavior arises from their hard and rigid nature. In the early sliding stages, glass fibers may protrude from the polymer matrix, causing mechanical abrasion on the steel ball, as illustrated in Figure 13.

Although glass fibers are harder than steel, they are embedded in an epoxy polymer matrix, this serves as a protective layer, minimizing direct contact between the fibers and the steel surface. This prevents the aggressive abrasive interactions typically responsible for severe metallic wear. Additionally, a thin transfer layer consisting of polymeric material from the GFRP forms on the steel ball’s surface during the test, further reducing friction and shielding the steel from direct abrasive action. This transfer layer minimizes the plowing effect of the fibers and helps stabilize the coefficient of friction (COF), which remains within a low range (~0.35–0.54), indicating the absence of severe adhesion or three-body abrasion.

Moreover, the variation in fiber weight fraction (50%, 65%, and 70%) within the GFRP does not significantly impact the wear behavior of the steel balls. Since the primary wear mechanisms in this system involve polymer matrix degradation and fiber pull-out, rather than direct fiber-to-steel abrasion, the steel balls remain largely unaffected in all tested cases. While increasing the fiber content strengthens the composite, the absence of direct fiber contact with the steel means that no notable increase in steel ball wear occurs, regardless of the fiber weight fraction.

In conclusion, the minimal wear of the 52100-steel balls across all test conditions is due to their intrinsic hardness and wear resistance, the cushioning effect of the polymer matrix, the development of a protective transfer layer, and the absence of severe wear mechanisms such as adhesion or deep abrasive plowing. These findings confirm that 52100-steel maintains its integrity in GFRP tribological applications, making it a suitable material for prolonged frictional use without significant degradation.

**Figure 14:**
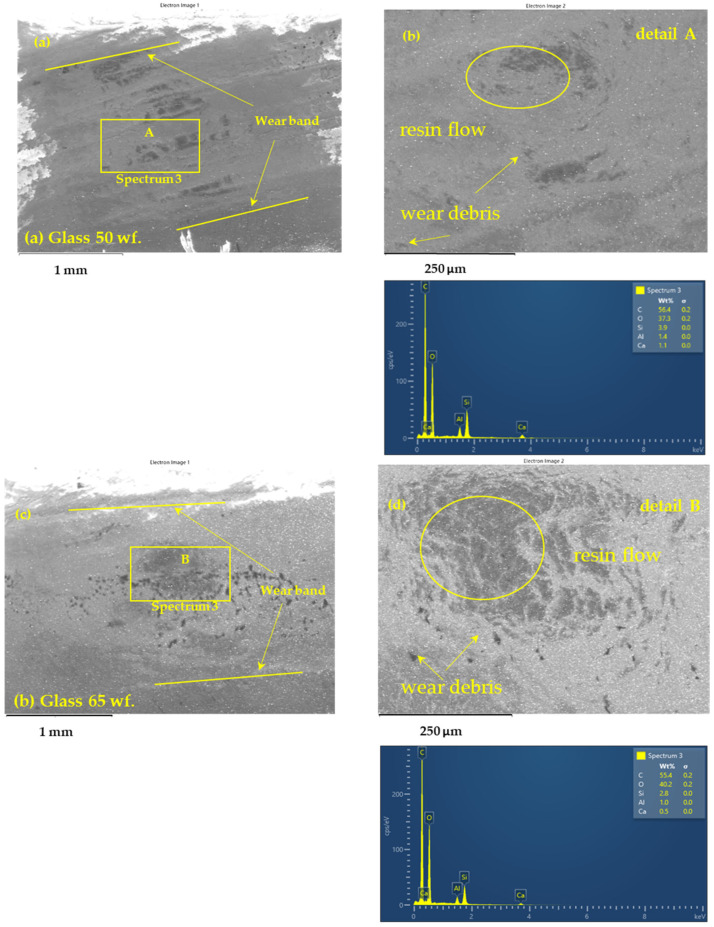

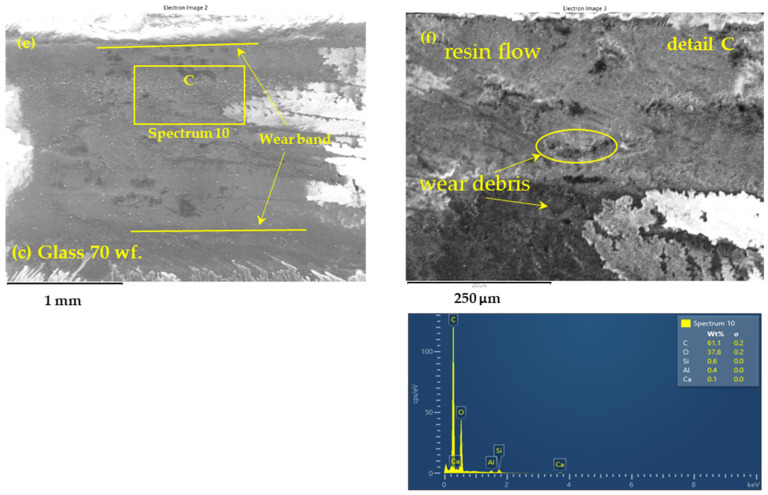
SEM images of wear tracks on GFRP samples after 120 min of dry sliding against a 52100-steel ball under a 30 N load and a 0.25 m/s sliding speed: (**a**,**b**) GFRP 50% overview and detailed wear track and EDS spectra; (**c**,**d**) GFRP 65% overview and detailed wear track and EDS spectra; and (**e**,**f**) GFRP 70% overview and detailed wear track and EDS spectra.

The SEM images presented in Figure 14 illustrate the microstructural modifications of the worn surfaces of GFRP samples (50%, 65%, and 70% wf.) after 120 min of testing under a 30 N load and a sliding speed of 0.25 m/s. These images highlight the differences in wear behavior of the composites, emphasizing the dominant mechanisms that contribute to material degradation.

In the case of the GFRP 50%, sample, prominent abrasive wear marks are observed, characterized by significant removal of the polymer matrix and exposure of the glass fibers. This sample exhibits the deepest wear marks, indicating lower friction resistance and accelerated degradation under load. In contrast, the GFRP 65% sample shows uniform wear marks with reduced material removal, suggesting a more stable interface between the epoxy matrix and glass fibers. The GFRP 70% sample presents a relatively smoother surface, indicating improved wear resistance and greater protection against abrasion.

The main identified wear mechanisms include micro cutting and the formation of fine grooves, which are more pronounced in the GFRP 50% sample, suggesting a higher material fragility. Delamination wear is evident in samples with a lower fiber content, where the composite’s surface layer separates more easily under frictional forces. In the GFRP 65% and 70% samples, the formation of a transfer layer is observed, which contributes to surface protection and reduces the coefficient of friction.

The local temperature in the contact area increases with the applied load, leading to localized softening of the polymer matrix under the influence of the high temperature generated by friction. As the matrix softens, it loses rigidity, which can result in reduced adhesion between the glass fibers and the epoxy matrix, promoting delamination wear and fiber extraction. However, in samples with a higher fiber content (70% wf.), the contact temperature is lower compared to the 50% and 65% samples due to more efficient heat dissipation through the fiber network. This observation correlates with a reduction in the coefficient of friction and the volume of material removed during the tribological tests.

Energy-dispersive X-ray spectroscopy (EDS) detected the presence of aluminum and silicon elements on the worn surfaces, with an increase in their concentration as the fiber content increases. In the case of the GFRP 50% sample, the surface layer contains smaller amounts of silicon and aluminum, suggesting faster wear of the polymer matrix. In contrast, for the GFRP 65% and 70% samples, the worn layer contains a higher proportion of these elements, indicating a protective effect of the glass fibers on the composite. The silicon detected in the EDS analysis primarily originates from the glass fibers of the GFRP composite, being released because of abrasive wear and tribological phenomena. Part of this silicon plays a crucial role in the formation of a protective layer on the contact surface, which explains the reduction in the friction coefficient at higher fiber contents (65–70%). The source of the aluminum detected in the wear layer is its natural presence in the glass fibers in the form of aluminosilicates.

The SEM and EDS results confirm that a higher fiber content improves the wear resistance of GFRP composites, reducing the coefficient of friction and dissipating thermal energy more efficiently during friction. Additionally, the microstructural analysis shows that abrasive wear is the dominant mechanism for GFRP 50%, while in GFRP 70%, the formation of a transfer layer enhances surface protection. These findings are crucial for optimizing the use of GFRP composites in tribological applications, where reducing friction and improving wear resistance are critical factors.

### 3.5. Interactions and Transformations Between Wear Mechanisms

The tribological behavior of GFRP composites in contact with 52100-chromium-alloyed steel balls is governed by multiple wear mechanisms that act concurrently. These mechanisms include abrasive wear, transfer layer formation, and polymer matrix degradation, each influenced by applied load, sliding speed, and fiber weight fraction. The transition between these mechanisms determines the wear performance and surface integrity of the composite material.

At low sliding speeds and high loads, abrasive wear is the dominant mechanism. The mechanical interaction between the steel ball and the composite leads to the removal of polymer matrix material, exposing the reinforcing glass fibers. However, as sliding speed increases, the frictional heat softens the polymer matrix, facilitating the formation of a transfer layer. This layer, consisting of polymeric material adhered to the steel counterface, acts as a protective barrier and reduces direct abrasive interactions, leading to a lower wear rate.

At very high sliding speeds, the accumulated heat can exceed the thermal stability of the polymer matrix, leading to localized softening or degradation. This weakens the interfacial bonding between fibers and the matrix, increasing the likelihood of fiber pull-out and adhesive wear. Additionally, thermal degradation can result in an unstable transfer layer, which may intermittently detach and expose fresh material to wear, creating fluctuations in the wear rate.

The interplay between these wear mechanisms is evident from SEM and EDS analyses, which reveal signs of both abrasion marks and polymer transfer layers. The transformation between these mechanisms is primarily dictated by the tribological parameters. At low loads and low speeds, minimal wear occurs, with limited fiber exposure and negligible polymer degradation. An optimal transfer layer is formed at moderate loads and sliding speeds, balancing wear resistance and reducing COF. At high loads and high speeds, polymer degradation intensifies, leading to increased fiber pull-out and fluctuating wear behavior.

The combination of abrasive, adhesive, and thermal effects explains the observed wear trends in the experimental results. These findings highlight the complex interactions between material properties and tribological conditions, providing valuable insights into the optimization of GFRP composites for frictional applications.

### 3.6. Correlation Between Structural Changes and Tribological Performance

The SEM and EDS analyses provide critical insights into the microstructural evolution of the worn surfaces and their direct impact on tribological behavior. The morphological differences observed among GFRP composites with varying fiber content reveal that higher fiber weight fractions contribute to lower polymer degradation and improved heat dissipation, ultimately enhancing wear resistance.

The EDS analysis of transfer layers further clarifies the role of material transfer in stabilizing frictional performance. The presence of silicon and aluminum elements from the glass fibers in the worn layers suggests that these components contribute to the formation of a protective surface layer. This helps reduce the coefficient of friction and mitigates excessive material removal.

Additionally, fiber pull-out and polymer degradation observed in SEM images indicate that at high loads and sliding speeds, weak fiber-matrix interfacial bonding accelerates surface damage, leading to increased wear. In contrast, composites with higher fiber content maintain structural integrity for longer durations, delaying wear initiation and improving overall tribological performance.

## 4. Conclusions

This study investigated the mechanical and tribological performance of twill-woven glass fiber-reinforced polymer (GFRP) composites with different fiber weight fractions (50%, 65%, and 70%). The research evaluated the influence of fiber content, applied load, and sliding speed on the coefficient of friction, specific wear rate, and contact temperature. Experimental results and statistical analysis provided an in-depth understanding of the wear mechanisms and the key factors influencing the tribological behavior of these materials.

The results demonstrate that the coefficient of friction is significantly influenced by sliding speed, which accounts for 46.51% of its variation. Additionally, the interaction between applied load and sliding speed had a considerable impact (32.51%), while the fiber weight fraction had a lower contribution (11%). Samples with a higher fiber content (70%) exhibited the lowest friction coefficient values, indicating improved wear resistance and reduced abrasive interaction at the contact surface. The maximum COF was obtained for a wf. of 50%, force of 30N, and v of 0.1m/s.

The analysis of specific wear indicated that fiber weight fraction is the most influential factor, contributing 34.15%, followed by applied load (21.87%) and sliding speed (14.11%). Wear decreased as the fiber content increased, confirming that a higher proportion of glass fibers enhances the material’s resistance to wear. In samples with 50% fibers, abrasive wear was more intense, whereas in those with 70% fibers, a protective effect was observed due to the formation of a transfer layer. The minimum K was obtained for a wf. of 70%, a force of 10 N, and v of 0.1 m/s.

Contact temperature was primarily influenced by applied load (39.08%) and the interaction between fiber fraction and sliding speed (27.24%). Temperature increases favored localized softening of the polymer matrix, leading to reduced adhesion between fibers and the epoxy matrix, thus promoting delamination wear. However, in composites with 70% fiber content, temperature was lower compared to 50% and 65% samples due to more efficient heat dissipation through the fiber network. This observation correlates with a reduction in the friction coefficient and specific wear rate.

SEM and EDS results confirmed that abrasive wear is the dominant mechanism in samples with 50% fibers, where pronounced micro-cutting and delamination marks were observed. In samples with 65% and 70% fibers, analysis revealed the presence of a transfer layer and an increase in aluminum and silicon concentrations on the worn surface, indicating a protective effect of the glass fibers. These observations support the conclusion that a higher fiber content contributes to stabilizing tribological interactions and improving the wear behavior of GFRP composites.

These findings are essential for optimizing the use of GFRP composites in industrial applications where friction and wear are critical factors, such as the aerospace, automotive, and mechanical engineering industries. Future studies should explore the long-term behavior of these materials under varying load and environmental conditions, as well as analyze the impact of other types of reinforcements on their tribological performance. Additionally, using predictive models based on machine learning could help optimize operating parameters and improve the durability of these advanced composites.

## Figures and Tables

**Table 1:** Laminated composite—name and composition.

Laminated Composite Symbolization	Fiber (%)	Epoxy Resin (%)
Laminated epoxy/glass fiber composite (wf. 50%)—GFRP_50	50	50
Laminated epoxy/glass fiber composite (wf. 65%)—GFRP_65	65	35
Laminated epoxy/glass fiber composite (wf. 70%)—GFRP_70	70	30

**Table 2:** Testing parameters.

Parameters	Operating Conditions
Load (Force)	10, 20, 30 N
Sliding Speed	0.1, 0.25, 0.36 m/s
Rotational Speed (RPM)	Max 215 (±3) rpm
Relative Humidity	40 (±5)%
Initial Temperature	20 (±2) °C
Test Duration	120 min
Conditions	Dry friction
Materials Disc and Ball	GFRP composites and 52100-chromium-alloyed carbon steel balls
Roughness of the Disc and Ball [Ra]	0.32 µm and 0.04 µm

**Table 3:** Control factors and their levels for statistical analysis.

Targets	Glass Fiber, wf.		Applied Load, F		Sliding Speed, v	
	Symbol	Value [%]	Symbol	Value [N]	Symbol	Value [m/s]
Coefficient of friction Specific wear rate Temperature	1	50	1	10	1	0.10
	2	65	2	20	2	0.25
	3	70	3	30	3	0.36

**Table 4:** Tensile properties of GFRP specimens.

Specimen	Tensile Strength [MPa]/ SD [MPa]/CV [%]	Tensile Strain at Maximum Load [%]/ SD [%]/CV [%]	Elastic Modulus E [MPa]/ SD [MPa]/CV [%]
GFRP 70%	480.1/(25.92)/5.39	2.94/(0.03)/1.02	22,181.7/(253.2)/1.14
GFRP 65%	376.57/(24.37)/6.47	2.63/(0.02)/0.76	18,720.6/(316.36)/1.73
GFRP 50%	318.8/(22.45)/7.04	2.41/(0.02)/0.82	14,602/(301.25)/2.06

SD—Standard Deviation; CV—Coefficient of Variation

**Table 5:** Flexural properties of GFRP specimens.

Specimen	Flexural Strength [MPa]/ SD [MPa]/CV [%]	Flexural Strain [%]/ SD [%]/CV [%]	Elastic Modulus E [MPa]/ SD [MPa]/CV [%]
GFRP 70%	464.8/(11.47)/2.46	3.77/(0.16)/4.24	21,009/(392)/1.52
GFRP 65%	415.5/(21.56)/5.18	3.08/(0.13)/4.22	18,218/(484)/2.65
GFRP 50%	388.6/(36.8)/9.46	2.36/(0.25)/9.59	14,935/(199)/2.94

SD—Standard Deviation; CV—Coefficient of Variation

**Table 6:** Experimental results of dry sliding wear.

Experimental Parameters				Mean of Measured Parameters		
Exp.nr.	Applied Load F [N]	Sliding Speed v [m/s]	Glass Fiber wf. [%]	Specific Wear Rate K [10^−5^ mm^3^ × (Nm)^−1^]	Coefficient of Friction [µ] Average of the Last 60 min	Temperature Average of the Last 60 min [°C]
1	10	0.1	50	5.758	0.54	32.7
2	10	0.1	65	3.42	0.5	29.4
3	10	0.1	70	1.5488	0.43	35.1
4	10	0.25	50	20.985	0.4	35
5	10	0.25	65	12.267	0.39	36.2
6	10	0.25	70	6.4267	0.38	31
7	10	0.36	50	14.907	0.39	40.2
8	10	0.36	65	16.022	0.38	41.4
9	10	0.36	70	9.59	0.34	25.1
10	20	0.1	50	15.208	0.54	32
11	20	0.1	65	14.189	0.48	29.2
12	20	0.1	70	11.456	0.41	35.9
13	20	0.25	50	24.906	0.45	47.5
14	20	0.25	65	32.737	0.43	45.7
15	20	0.25	70	6.12	0.42	37.8
16	20	0.36	50	26.019	0.37	48.7
17	20	0.36	65	23.291	0.36	51.7
18	20	0.36	70	4.42	0.35	46.3
19	30	0.1	50	21.671	0.51	40.8
20	30	0.1	65	18.256	0.49	34.9
21	30	0.1	70	14.426	0.48	62.7
22	30	0.25	50	32.385	0.37	52.4
23	30	0.25	65	23.727	0.36	52.7
24	30	0.25	70	14.341	0.33	47.1
25	30	0.36	50	14.488	0.49	61.3
26	30	0.36	65	19.191	0.48	62.7
27	30	0.36	70	10.492	0.47	34.2

**Table 7:** The statistical results for coefficient of friction, specific wear rate, and temperature.

		COF			K			T	
Source	F-Value	*p*-Value	PC [%]	F-Value	*p*-Value	PC [%]	F-Value	*p*-Value	PC [%]
wf	18.17	0.001	11	17.08	0.001	34.15	0.84	0.466	2.71
F	5.07	0.038	3.07	10.94	0.005	21.87	12.13	0.004	39.08
v	76.85	<0.001	46.51	7.05	0.017	14.11	3.85	0.068	12.39
wf*F	0.81	0.551	0.98	1.47	0.296	5.89	0.06	0.992	0.4
wf*v	2.90	0.094	3.51	2.07	0.177	8.28	4.23	0.039	27.24
F*v	26.86	<0.001	32.51	1.92	0.200	7.7	0.82	0.546	5.3
Error	-	-	2.42	-	-	8	-	-	12.88
Total	-	-	100	-	-	100	-	-	100

Note: “*” signifies the interaction between factors.

## Data Availability

Data are contained within the article.
